# Editorial: The 13th annual meeting of the Italian chapter of the European society of biomechanics: exploring new insights in the biomechanics field through innovative and advanced approaches

**DOI:** 10.3389/fbioe.2026.2030574

**Published:** 2026-10-29

**Authors:** Cristina Falcinelli

**Affiliations:** Department of Engineering and Geology, University G d’Annunzio Chieti-Pescara, Pescara, Italy

**Keywords:** biomaterials, biomechanics, computational modeling, experimental test, medical devices

Bioengineering is advancing our understanding of how healthy and pathological tissues respond to mechanical stimuli and how their interactions with medical devices can be optimized. Progress in imaging, experimental testing, computational modeling, biomaterials, and advanced manufacturing has opened new opportunities to characterize biological systems and translate this knowledge into improved diagnosis and treatment. Integrating these approaches is important for capturing the complex relationships between tissue structure, mechanical properties, and function.

Arising from the $13^{\text{th}}$ Annual Meeting of the Italian Chapter of the European Society of Biomechanics, held in Pescara from 3 to 4 October 2024, this Research Topic highlights recent progress and emerging trends in biomechanics and bioengineering, illustrating how innovative approaches can give a more in-depth knowledge of healthy and pathological tissues and support the development of clinically relevant solutions.

The articles in this Research Topic address different aspects of biomechanics and highlight the importance of mechanical characterization in investigating biological processes and clinically relevant questions. Three contributions address dental and craniofacial systems. In detail, at the cellular level Putame et al. developed a bioreactor platform for applying controlled intermittent stretching to human periodontal ligament stem cells. By characterizing deformation of the cell substrate and comparing responses across stretching protocols, they showed that greater daily cyclic exposure was associated with cell alignment and increased expression of osteogenic and periodontal-ligament-related genes. Adipose-derived stem cells did not show the same response under the tested conditions, underscoring the importance of tissue-specific mechanobiology. At the tissue level, Stróżyk used digital image correlation (DIC) to map temporalis muscle surface displacement *in vivo* during unilateral chewing. In a preliminary study of eight healthy men, the measurements revealed distinct regional kinematic patterns and showed that local motion varied with chewing side and food texture. These findings support the use of non-contact, full-field measurements to investigate muscle function during physiological tasks. At the organ level, Becker et al. compared subject-specific mandibular finite element predictions with experimental measurements using eight density-elasticity relationships. The study showed that predictions varied substantially depending on the density–elasticity relation used, and that none of the relationships consistently reproduced the experimental response, particularly the local surface strain. This finding highlights how constitutive assumptions affect model predictions and the need for experimental validation and sensitivity analysis.

Biomechanical approaches were also applied to respiratory, urinary, and cardiovascular systems. Huang et al. combined tensile tests of human lung bullae and parenchyma with computed tomography (CT)-derived, geometry-specific finite element models. Although bullae showed higher tensile strength than adjacent parenchyma, computed stress concentrations at the bulla apex offer a plausible mechanical explanation for the clinical tendency toward apical rupture. Liu et al. paired transvaginal ultrasound with peristaltic analysis to quantify ureteral wave characteristics in healthy women. Their pilot study demonstrated the feasibility of non-invasive measurement of dynamic parameters, including wave speed and transmission distance, and provides a basis for testing the method in patients with ureteral disorders. Finally, Mazzoli et al. combined statistical shape modeling and computational fluid dynamics to examine how aortic morphology affects the watershed zone during veno-arterial extracorporeal membrane oxygenation (VA-ECMO) support. Their simulations indicate that both aortic geometry and ECMO flow level influence the zone’s position and distribution, highlighting anatomical variability as a factor in hemodynamic assessment.

The six contributions share a quantitative approach to linking mechanical behavior with biological or clinical function. Their methods span mechanical testing, bioreactor experiments, DIC, ultrasound-based motion analysis, CT-based geometric reconstruction, subject-specific finite element analysis, statistical shape modeling, and computational fluid dynamics. Across these systems, the studies investigate how tissue properties, geometry, loading, and measurement choices influence the observed mechanical or biological response. The mandibular study highlights the sensitivity of model predictions to constitutive assumptions; the lung and VA-ECMO studies show that anatomy can influence local mechanics and flow; and the dental cell and muscle studies link mechanical stimulation or movement patterns to biological function. The ureter study extends this approach toward functional, non-invasive assessment.

Future advancements will depend on validation in larger cohorts and clinically relevant conditions, refinement of material laws and boundary assumptions, and connection of quantitative mechanical measures to patient outcomes and device performance. Taken together, the studies in this Research Topic suggest that combining experimental, imaging-based, and computational approaches can provide a more comprehensive understanding of biomechanical mechanisms and support clinical decisions that account for individual patient characteristics.

